# Antenatal corticosteroids for neonates born before 25 Weeks—A systematic review and meta-analysis

**DOI:** 10.1371/journal.pone.0176090

**Published:** 2017-05-09

**Authors:** Mangesh Deshmukh, Sanjay Patole

**Affiliations:** 1 Department of Neonatal Pediatrics, St. John of God Hospital, Subiaco, Perth, Western Australia; 2 Department of Neonatal Pediatrics, Fiona Stanley Hospital, Perth, Western Australia; 3 Department of Neonatal Pediatrics, King Edward Memorial Hospital, Perth, Western Australia; 4 Centre for Neonatal Research and Education, University of Western Australia, Perth, Western Australia; Hopital Robert Debre, FRANCE

## Abstract

**Background:**

Efficacy of antenatal corticosteroids before 25 weeks of gestation is unclear.

**Objective:**

To assess and compare neonatal outcomes following ANC exposure at 22, 23 and 24 weeks of gestation by conducting systematic review and meta- analysis.

**Methods:**

A systematic review of randomised controlled trials (RCT) and non-RCTs reporting on neonatal outcomes after exposure to ANC up to 24^6^ weeks of gestation using the Cochrane systematic review methodology. Databases Pubmed, CINAHL, Embase, Cochrane Central library, and online abstracts of conference proceedings including the Pediatric Academic Society (PAS) were searched in Feb 2017. Primary outcome was in-hospital mortality defined as death before discharge during the first admission. Secondary outcomes included severe intraventricular hemorrhage (IVH> grade III and IV)/or periventricular leukomalacia (PVL), necrotising enterocolitis (NEC >stage II) and chronic lung disease (CLD). Meta-analysis was performed using a random-effects model. The level of evidence (LOE) was summarised using the GRADE guidelines.

**Main results:**

There were no RCTs; 8 high quality non-RCTs were included in the review. Meta-analysis showed reduction in mortality [N = 10109; OR = 0.47(0.39–0.56), p<0.00001; LOE: Moderate] and severe IVH and PVL [N = 5084; OR = 0.71(0.61–0.82), p<0.00001; LOE: Low] after exposure to ANC in neonates born <25 weeks. There was no significant difference in CLD [N = 4649; OR = 1.19(0.85–1.65) p = 0.31; LOE: Low] and NEC [N = 5403; OR = 0.95 (0.76–1.19) p = 0.65; LOE: Low]. Mortality was comparable in neonates born at 22, 23 or 24 weeks.

**Conclusion:**

Moderate to low quality evidence indicates that exposure to ANC is associated with reduction in mortality and IVH/or PVL in neonates born before 25 weeks.

## Introduction

Administering antenatal corticosteroids (ANC) for impending preterm delivery before 34 weeks’ of gestation is a standard practice. A systematic review of randomised controlled trials (RCT) indicates that exposure to ANC significantly reduces neonatal mortality and morbidity such as respiratory distress, cerebrovascular haemorrhage, and necrotising enterocolitis (NEC).[1] The guidelines of Obstetric and Gynaecological Colleges/Societies recommend ANC between 24–34 weeks of gestation. [2–5] The updated guidelines of the Royal College of Obstetricians and Gynaecologist (RCOG), the consensus statement from the American College of Obstetricians and Gynaecologist (ACOG), and the New Zealand-Australia guidelines suggest ANC should be considered from 23 weeks’ gestation after careful evaluation of the benefit and risks with parenteral consultation. [2, 4, 5]

There is a broad consensus for not offering active management including ANC and neonatal resuscitation for delivery <23 weeks’.[6]A review of the international guidelines for management of extremely preterm births showed an agreement for offering ANC, neonatal resuscitation, and intensive care after 25 weeks' gestation. [6] On the other hand the consensus statement from New South Wales, Australia suggests that it is acceptable not to initiate intensive care in neonates born at 22−25^6^ weeks’ gestation if parents wish so after appropriate counselling. [7] It is important to note that the evidence supporting ANC at <26 weeks’ gestation is based mainly on laboratory studies and non-RCTs. The only RCT (n = 49) in this field is from the pre-surfactant era. [8] The RCOG and ACOG guidelines acknowledge this limitation. [2, 4]

Given the lack of clarity on guidelines for ANC at extremely preterm gestations there is significant variation in clinical practice for managing such pregnancies. [9, 10] For example in the Express cohort study from Sweden, a high percentage of neonates (23 weeks: 85%, 24 and 25 weeks: > 95%) were exposed to ANC, whereas in the EPIPAGE-2 cohort study from France only 12.3% of neonates at 23 weeks, 56.7% at 24 weeks and 78.4% at 25 weeks gestation were given ANC. [11] A recent systematic review has addressed the issue of ANC for impending deliveries at 22 and 23^6^ weeks’ gestation.[10] Meta-analysis of 4/17 included non-RCTs indicated that the adjusted odds of mortality to discharge (Primary outcome) were reduced by 52% in the ANC vs. control group. Severe morbidity was not significantly different between the two groups.[12]

Overall, the data supporting ANC are scarce for neonates born at 22–26 weeks' gestation. At 25 weeks of gestation most will offer ANC after parental counselling. However, controversy continues about ANC not only at 22 and 23 weeks, but also at 24^0−6^ weeks of gestation. Comparing neonatal outcomes between 22, 23, and 24 weeks’ gestation following ANC exposure is important because not offering this intervention at these gestations may mean increased risk of death and/or survival with significant morbidity. Given the mortality, morbidity and potential benefits of ANC at such very early gestations, we conducted a systematic review comparing neonatal outcomes following ANC exposure at 22, 23 and 24 weeks of gestation. The data is expected to be useful for parental counselling and decision making about neonatal management at these gestations.

## Materials and methods

The Cochrane methodology and MOOSE guidelines (Meta-analysis of Observational Studies in Epidemiology) were used for conducting and reporting this systematic review respectively. [13, 14] Ethics approval was not required.

### Eligibility criteria

#### Types of studies

Randomised controlled trials **(**RCTs), quasi-RCTs and non-RCTs were eligible for inclusion. Reviews and commentaries were excluded, but read to identify other potential studies

#### Participants

**Inclusion criteria:** Neonates born <25 (22–24^+6^) weeks of gestation.

**Exclusion criteria:** Major chromosomal and congenital anomalies.

#### Type of intervention

Antenatal glucocorticosteroids of any type (e.g. Betamethasone, Dexamethasone), dose (single/multiple), duration vs. placebo/control.

#### Types of outcomes

**Primary outcome:** 1) Mortality: Death before discharge from the neonatal unit during the first admission after birth.

**Secondary outcomes: (**1) Severe intraventricular haemorrhage (IVH> grade III and IV)/or periventricular leukomalacia (PVL)[15] (2) Necrotising enterocolitis (NEC: ≥ Stage II)[16] (3) Chronic lung disease (CLD) defined as requirement of oxygen at 36 weeks.[17]

#### Search strategy

We searched MEDLINE (from 1946), EMBASE (from1974), CINAHL and Cochrane Central register of Controlled Trials initially in November 2016 and updated in February 2017 for published studies. We used the following search terms in various combinations: a) Population- neonate(s), newborn(s), infant*, premature, very low birth weight, extremely low birth weight (s), b) Intervention- antenatal corticosteroids, adrenocortical stimulating hormone, Betamethasone, Celestone, Dexamethasone, c) Outcome- mortality, intraventricular haemorrhage, NEC and publication type “Randomized controlled Trial, “Controlled Trial”, or “Clinical Trial”, Cohort, Case control studies. Online abstracts of Pediatric Academic Society (PAS) meetings were reviewed from 2002. Abstracts of conference proceedings including Perinatal Society of Australia and New Zealand (PSANZ), European academy of Paediatric societies, and the British Maternal and Fetal Medicine Society were searched in EMBASE. ‘Google Scholar’ was searched for articles that might not have been cited in the standard medical databases. The reference lists of identified studies and review articles were searched to identify additional eligible studies. We also searched http://www.clinicaltrials.gov and Australian New Zealand trial registry (http://www.anzctr.org.au) for ongoing studies. No language restriction was applied. Reviewers MD, SP and librarian (Ms Marcia Powell) at our institute conducted the literature search independently.

#### Study selection

The abstracts of the citations obtained from the initial broad search were read independently by reviewers MD and SP, to identify potentially eligible studies. Full text articles of these studies were obtained and assessed independently by reviewers MD and SP for eligibility using the predefined eligibility criteria. Differences in opinion were resolved by group discussion among all reviewers to reach consensus. Multiple publications of the same study were identified and excluded, to avoid duplication of the data.

#### Data extraction

Reviewers MD and SP extracted the data independently, using a data collection form designed for this review. For dichotomous outcomes, the number of patients with the event and the number of patients analysed in each treatment group of each trial were entered into the form. For continuous outcomes, we planned to enter the mean and standard deviations. Information about study design and outcomes were verified by both reviewers. We also checked specifically for adjustment of data for maternal and neonatal confounding factor in non RCTs. Discrepancies during the data extraction process were resolved by discussion and consensus among both the reviewers. We contacted authors for additional information/clarifications when details were not available in published manuscripts.

#### Risk of bias assessment

Risk of bias for RCTs and quasi RCTs was assessed using the Cochrane “Risk of Bias Assessment Tool”.[18] The assessments were done independently by two reviewers in the domains of random sequence generation, allocation concealment, blinding of participants and outcome assessors, completeness of follow up, selective reporting and other biases. The studies were assigned as carrying high, low or unclear risk of bias. The quantitative scoring tool, Newcastle-Ottawa scale (NOS), proposed by Cochrane Collaboration, was adopted for evaluating the methodological quality of the included non-RCTs.[19] The NOS contains three major domains: selection of subjects, comparability between groups, and the outcome measures. The maximum score for each area is four, two and three points respectively. A total score of three or lower indicates low methodological quality.

#### Data synthesis

Meta-analysis was performed using Review manager 5.3 [Cochrane Collaboration, Nordic Cochrane Centre] if pooling of data was possible and justified with ‘intention to treat analysis’ of the data. We used a random-effects model assuming wide heterogeneity. Categorical measure of effect size was expressed as odds ratio (OR) (Mantel Haenszel method). Statistical heterogeneity was assessed using the Chi-Squared test, I^**2**^ statistic, and by visual inspection of the forest plot (overlap of confidence intervals). A narrative synthesis was planned if meta-analysis was not possible due to significant heterogeneity in included studies and/or non-availability of the outcome measures in the desired form. Sensitivity analysis was planned excluding the studies where the data was not adjusted for maternal and neonatal variables.

#### Assessment of publication bias

We assessed publication bias using funnel plot which comprises of odds ratio (effect size) plotted on the x axis and the standard error on the y axis. The typically symmetrical funnel plot shows studies with larger sample size at the top clustering around the mean effect size (midline) whereas those with smaller sample size arespread around the broad range of values.[20]

#### Summary of findings

The data concerning the quality of evidence, the magnitude of effect of the intervention, and the sum of available data on the main outcomes were presented in the ‘Summary of findings table’ as per the GRADE (The Grading of Recommendations Assessment, Development and Evaluation) guidelines. [21]

## Results

**Fig 1:**
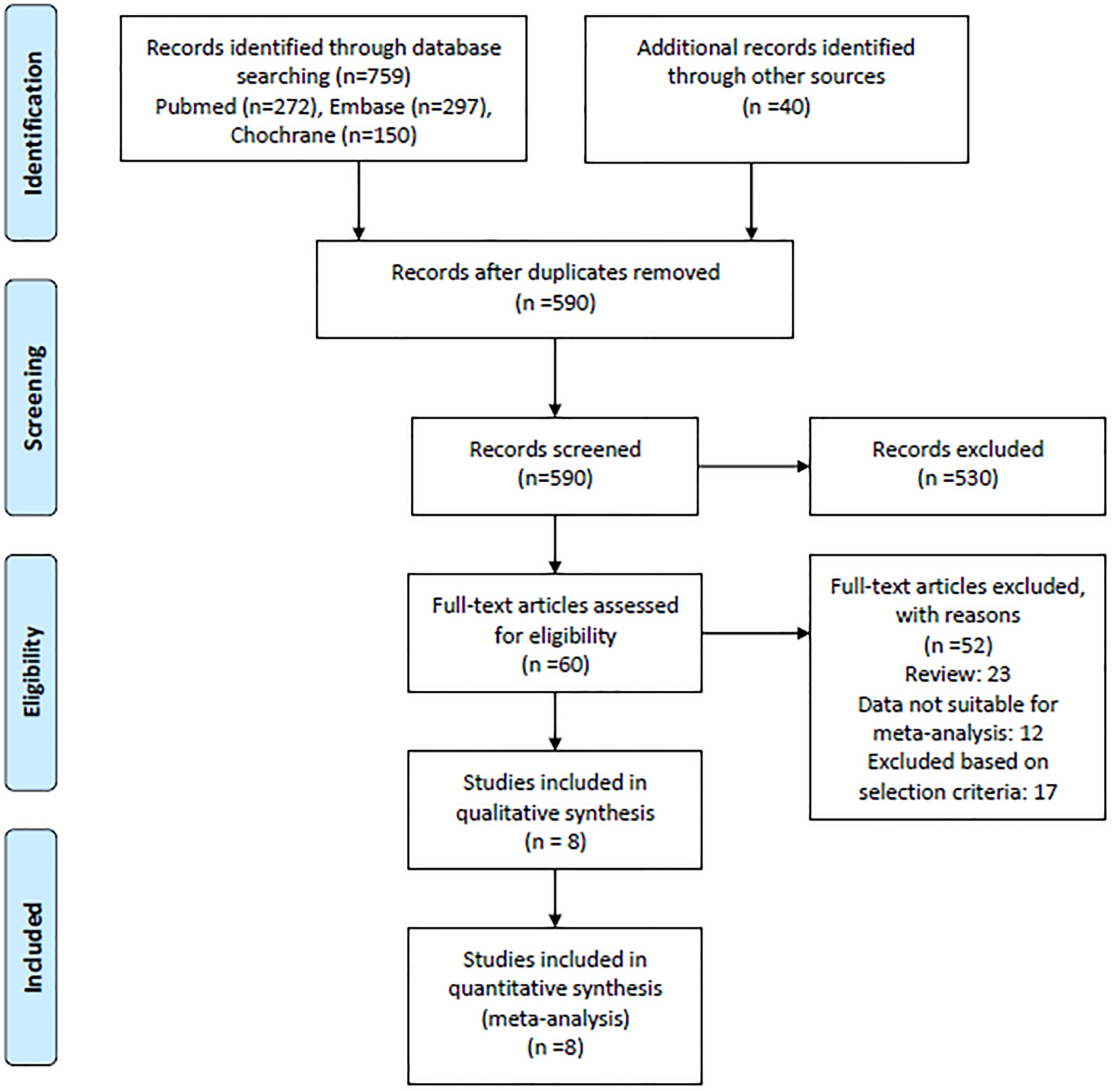
Flow chart of study selection process after screening electronic search.

The literature search retrieved 1368 potential relevant citations (Fig 1). After review of the abstracts and titles 1020 citations were excluded as they were not relevant. A total of 288 citations were identified as duplicate and excluded. Total 60 citations were read in detail with another 40 excluded for reasons mentioned in the flow chart. There were no RCT fulfilling the inclusion criteria. A total of 20 non-RCTs were eligible for inclusion in the systematic review. Data from 12 of these 20 studies was not available for meta-analysis despite contacting the authors. Finally, 8 non-RCTs were included in the review. [22–29] Total 5/8 studies provided details on the type of glucocorticosteroids (betamethasone, dexamethasone or hydrocortisone) [24, 26–29] used. The remaining 3 studies didn’t provide these details.[22, 23, 25] The large studies by Carlo et al and Mori et al provided data for all the outcomes included in the review at all the gestation[24, 28]. Guthrie et al provided the data only for the outcome of NEC.[29] We obtained additional data on request from authors Mori et al, Bajwa et al and Guinsburg et al.[23, 25, 28] We used the systematic review by Park et al for the data on Bader et al and Guthrie et al as we were unable to get it from the original authors.[12, 22, 29] Only 2 studies reported on long-term neurodevelopment at the corrected age of 12–24 months.[23, 24] The characteristics and quality assessment of the included studies are described in Table 1. These studies enrolled total 10109 neonates and had significant variation in treatment type, dose and duration. Data was adjusted for maternal and neonatal variables for most of included studies except Manktelow et al.[27]

**Table 1:** Characteristics and quality of included studies.

Study	Gest weeks	Design	Steroid type	Primary outcomes	Adjusted variables	Quality of study NOS^#^		
						Selection	Comparability	Outcome
Carlo 2011	22–25	Pros.	Beta or Dexa	Mortality and neurodevelopmental impairment at 18–22 months	Maternal age, hypertension or preeclampsia, rupture of membranes >24 hrs and delivery mode, multiple births, neonatal gender, and centre.	****	**	***
Hayes 2008	23	Retro.	Beta or Dexa	Neonatal mortality	Maternal age, race, plurality, antibiotic administration at delivery, route of delivery, and neonatal gender and birth weight.	****	**	***
Manktelow 2009	23–32	Pros.	Beta or Dexa	Neonatal mortality	Not adjusted	****	*	***
Mori 2011	22–33	Retro.	Beta or Dexa	Respiratory distress, Mortality, CLD, IVH and NEC.	Maternal age, parity, multiplicity, GDM, hypertension, PROM, mode of and place of delivery, neonatal gender, birth weight.	****	*	***
Guinsburg 2016	23–33+6	Pros.	Not mentioned	Unfavourable neonatal outcome	Maternal and neonatal morbidity variables	****	**	***
Bajwa 2011	23–32	Pros.	Not mentioned	Neonatal mortality	Neonatal gender and growth restriction.	****	**	***
Guthrie^¶^ 2003	23–34	Retro.	HC or Dexa	NEC	Neonatal birth weight, Apgar score at 5 minutes, caesarean delivery, exposure to indomethacin	****	**	***
Bader^¶^ 2010	23–26	Pros.	Not mentioned	Neonatal mortality	Maternal ethnicity, infertility treatment, plurality, hypertensive disorders amnionitis, neonatal birth weight and gender.	****	**	***

#NOS: New castle Ottawa scale.

*: The number of stars indicate score as per NOS.

^¶^Data from Park et al.

Pros- Prospective, Retro- Retrospective, Beta- Betamethasone, Dexa- Dexamethasone, HC- Hydrocortisone- Chronic lung disease, NEC- Necrotising enterocolitis, IVH- Intraventricular Haemorrhage, PVL- Periventricular Leukomalacia, GDM- Gestational diabetes mellitus, PROM- Prolonged rupture of membrane.

### Primary outcome

#### Mortality (Gestation <25 weeks)

**Fig 2:**
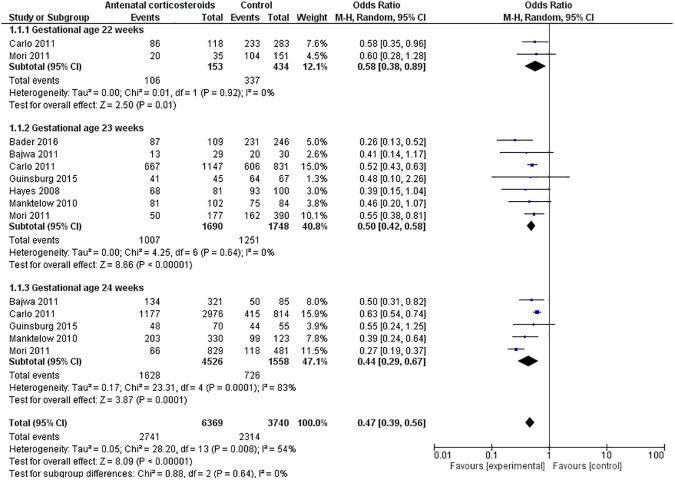
Effect of antenatal corticosteroids on neonatal mortality.

The data for mortality was available from 7 studies that included 10109 neonates (ANC: 6369, Control: 3740) born between 22–24 weeks’ gestation.[22–28] Mortality was significantly less in the ANC vs. control group neonates [ANC: 43% vs. Control: 62%]. Meta-analysis confirmed these findings [OR = 0.47(0.39–0.56) p<0.00001; Heterogeneity: Chi^2^ = 28.20, I^2^ = 54%)] (Fig 2). Sensitivity analysis performed excluding Manktelow et al as pre stated (data not adjusted for maternal and neonatal variable) didn’t change the results for <25 weeks as whole and individually for each gestation. The results by gestational age are as follows:

#### Mortality (Gestation 22–22^+6^ weeks)

Only two studies reported this outcome (N = 587). [24, 28] The individual studies showed statistically significant reduction in mortality in the ANC vs. control group. Meta-analysis showed that ANC was associated with significantly low mortality in neonates born at 22 weeks of gestation. [OR = 0.58(0.38–0.89), p = 0.01; Heterogeneity: Chi^2^ = 0.01, I^2^ = 0%)].

#### Mortality (Gestation 23–23^+6^ weeks)

A total seven studies reported this outcome (N = 3438). [22–28]All showed trend towards reduction of mortality in ANC group as compared to the control with 3/7 reaching statistically significant effect size (N = 2900).[22, 24, 28] The overall pooled estimate suggested antenatal corticosteroid was associated with significantly less mortality in neonates born at 23 weeks of gestation [OR = 0.50(0.42–0.58) p< 0.00001; Heterogeneity: Chi^2^ = 4.25, I^2^ = 0%)].

#### Mortality (Gestation 24–24^+6^ weeks)

Five studies reported this outcome (N = 6084).[23–25, 27, 28] Four out of five studies reported statistically significant reduction in mortality at this gestational age while study by Guinsburg et al (N = 125) showed trend towards reduction in mortality[25]. The overall pooled estimate suggested antenatal corticosteroid was associated with significant reduction in mortality in neonates born at 24 weeks of gestation. [OR = 0.44(0.29–0.67) p<0.0001; Heterogeneity: Chi^2^ = 23.31, I^2^ = 83%)].

### Secondary outcomes

#### 1. Severe IVH or PVL (Gestation <25 weeks)

**Fig 3:**
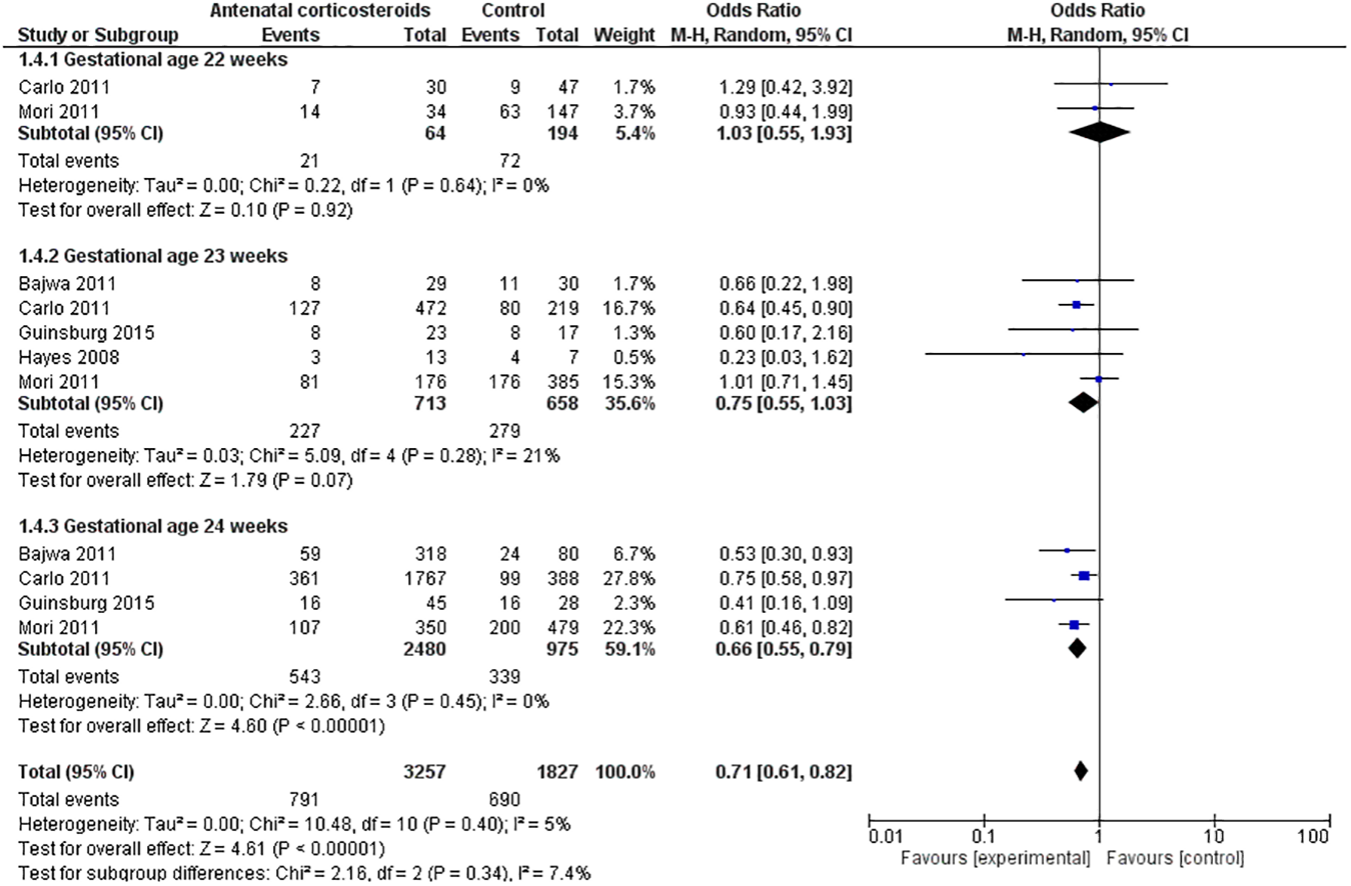
Effect of antenatal corticosteroids on severe IVH/or PVL.

The data for severe IVH or PVL was available from 5 studies that included 5084 neonates (ANC = 3257, Control = 1827) born between 22–24 weeks’ gestation.[23–26, 28] Severe IVH or PVL was significantly less in the ANC vs. control group neonates [ANC: 30% vs. Control: 38%]. Meta-analysis confirmed these findings [OR = 0.71(0.61–0.82) p< 0.00001; Heterogeneity: Chi^2^ = 10.48, I^2^ = 5%)]. (Fig 3) The results by gestational age are as follows:

**Severe IVH or PVL (Gestation 22–22**^**+6**^**)**: Only two studies reported this outcome (N = 258). [24, 28] The individual studies showed no reduction in IVH/or PVL in ANC vs. control group. The pooled estimate suggested no difference in IVH/or PVL between the two groups at 22 weeks of gestation. [OR = 1.03(0.55–1.93) p = 0.92; Heterogeneity: Chi^2^ = 0.22, I^2^ = 0%)].

**Severe IVH or PVL (Gestation 23–23**^**+6**^**)**: Five studies reported this outcome (N = 1371).[23–26, 28] Apart from Carlo et al no study showed difference in IVH/or PVL in the two groups[24]. The pooled estimate suggested no difference in incidence of IVH/or PVL in the two groups at 23 weeks of gestation [OR = 0.75(0.55–1.03) p< 0.07; Heterogeneity: Chi^2^ = 5.09, I^2^ = 21%)].

**Severe IVH or PVL (Gestation 24–24**^**+6**^**)**: Four studies reported this outcome (N = 3455).[23–25, 28] Three studies reported significant reduction in IVH/or PVL.[23, 24, 28] Meta-analysis showed that ANC was associated with significantly less IVH/or PVL in neonates born at 24 weeks of gestation [OR = 0.66(0.55–0.79) p< 0.0001; Heterogeneity: Chi^2^ = 2.66, I^2^ = 0%)].

#### 2. CLD (Gestation <25 weeks)

The data for CLD was available from 5 studies that included 4649 neonates (ANC = 3197, Control = 1452) born between 22–24 weeks’ gestation.[23–25, 27, 28] The overall incidence was 72% vs 68.7% between ANC vs Control arm. The Meta-analysis suggested no difference in CLD in two groups [OR = 1.19(0.85–1.65) p = 0.31; Heterogeneity: Chi^2^ = 27.09, I^2^ = 59%)]. (S1 Fig) Sensitivity analysis performed excluding Manktelow et al didn’t change the results for < 25 weeks as a whole and for individual gestation. The results by gestational age are as follows:

**CLD (Gestation 22–22**^**+6**^**)**: Only two studies reported this outcome (N = 144).[24, 28] The individual studies showed no statistically significant reduction in CLD. The pooled estimate suggested no difference in CLD between the two groups at 22 weeks of gestation. [OR = 1.19 (0.52–2.73) p = 0.68; Heterogeneity: Chi^2^ = 0.23, I^2^ = 0%)].

**CLD (Gestation 23–23**^**+6**^**)**: Five studies reported this outcome (N = 1136).[23–25, 27, 28] All studies showed no difference in CLD in the two groups. The meta- analysis also suggested no difference in incidence of CLD in the two groups at 23 weeks of gestation. [OR = 0.94(0.59–1.51) p = 0.81; Heterogeneity: Chi^2^ = 5.58, I^2^ = 28%)].

**CLD (Gestation 24–24**^**+6**^**)**: Five studies reported this outcome (N = 3369).[23–25, 27, 28] No studies reported reduction in CLD. The overall pooled estimate suggested no difference in incidence of CLD in the two groups at 24 weeks of gestation. [OR = 1.36 (0.86–2.17) p = 0.19; Heterogeneity: Chi^2^ = 12.09, I^2^ = 67%)].

#### 3. NEC (≥Stage II) (Gestation <25 weeks)

The data for NEC was available from 6 studies that included 5403 neonates (ANC = 3435, Control = 1968) born between 22–24 weeks’ gestation.[23–26, 28, 29] The overall incidence was 7.3% vs 7.8% between ANC vs Control arm. The Meta-analysis suggested no difference in NEC in two groups [OR = 0.95(0.76–1.19) p = 0.65; Heterogeneity: Chi^2^ = 8.82, I^2^ = 0%)]. (S2 Fig) The results by gestational age are as follows:

**NEC (Gestation 22–22**^**+6**^**weeks)**: Only two studies reported this outcome (N = 263).[24, 28] The individual studies showed no reduction in NEC. The pooled estimate suggested no difference between the two groups at 22 weeks of gestation. [OR = 0.59 (0.03–12.03) p = 0.73; Heterogeneity: Chi^2^ = 3.58, I^2^ = 72%)].

**NEC (Gestation 23–23**^**+6**^ **weeks)**: Six studies reported this outcome (N = 1601).[23–26, 28, 29] All studies showed no difference in NEC in the two groups. The pooled estimate confirmed no difference in incidence of NEC between the two at 23 weeks of gestation [OR = 0.93(0.66–1.32) p = 0.69; Heterogeneity: Chi^2^ = 4.82, I^2^ = 0%)].

**NEC (Gestation 24–24**^**+6**^ **weeks)**: Four studies reported this outcome (N = 3539).[23–25, 28] No studies reported reduction in NEC. The overall pooled estimate suggested ANC was not associated with reduction in NEC in neonates born at 24 weeks of gestation [OR = 0.95(0.71–1.29) p = 0.76; Heterogeneity: Chi^2^ = 0.86, I^2^ = 0%)].

#### Publication bias

**Fig 4:**
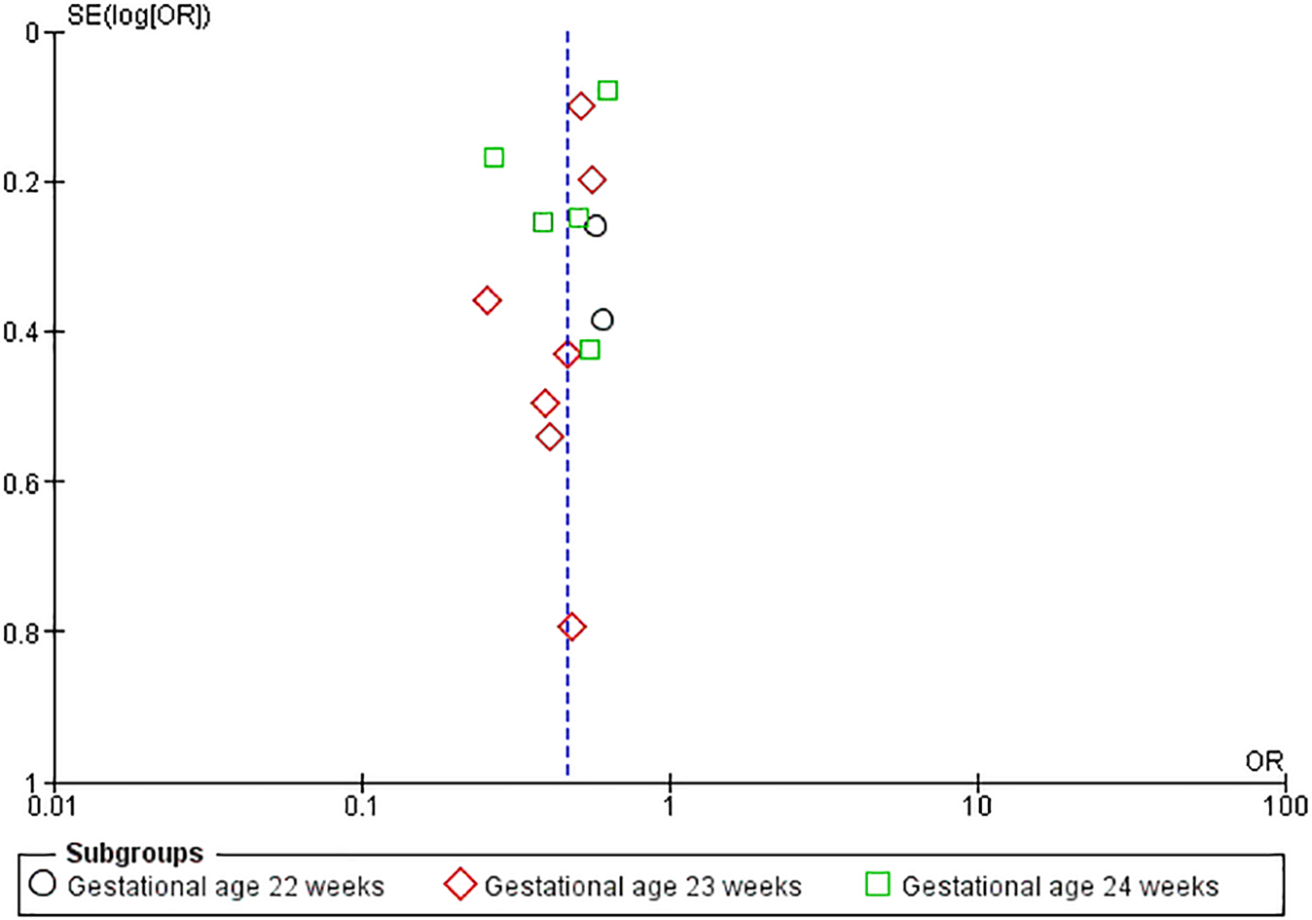
Funnel plot assessing publication bias.

Publication bias could not be ruled out as the number of included studies was small. [30] (Fig 4)

#### Grading of evidence and summary of findings

The quality of evidence was deemed as low for all outcomes considering the non-RCT design of the included studies. Evidence was upgraded for the outcome of mortality in view of very large effect size.[21] (Table 2).

**Table 2:** Summary of finding for pooled data as per GRADE guidelines.

Outcome	Gestational age	Absolute risk		Relative effect OR (95% CI)	Number of participants	Quality of evidence GRADE	Comment
		Estimated risk in control group	Corresponding risk in intervention group				
**Mortality**	22–24 weeks	619 per 1,000	**433 per 1,000** (388 to 476)	**OR 0.47** (0.39 to 0.56)	10109 (7 studies)	Moderate	#See below
	22 weeks	776 per 1,000	**668 per 1,000** (569 to 756)	**OR 0.58** (0.38 to 0.89)	587 (2 studies)	Moderate	#See below
	23 weeks	716 per 1,000	**557 per 1,000** (514 to 593)	**OR 0.50** (0.42 to 0.58)	3438 (7 studies)	Moderate	#See below
	24 weeks	466 per 1,000	**277 per 1,000** (202 to 369)	**OR 0.44** (0.29 to 0.67)	6084 (5 studies)	Moderate	#See below
**IVH (Stage III and IV) or PVL**	22–24 weeks	378 per 1,000	**301 per 1,000** (270 to 332)	**OR 0.71** (0.61 to 0.82)	5084 (5 studies)	Low	*See below
	22 weeks	371 per 1,000	**378 per 1,000** (245 to 532)	**OR 1.03** (0.55 to 1.93)	258 (2 studies)	Low	*See below
	23 weeks	424 per 1,000	**356 per 1,000** (288 to 431)	**OR 0.75** (0.55 to 1.03)	1371 (5 studies)	Low	*See below
	24 weeks	348 per 1,000	**260 per 1,000** (227 to 296)	**OR 0.66** (0.55 to 0.79)	3455 (4 studies)	Low	*See below
**CLD**	22–24 weeks	687 per 1,000	**723 per 1,000** **(651 to 783)**	**OR 1.19** **(0.85 to 1.65)**	4649 (5 studies)	Low	*See below
	22 weeks	750 per 1,000	**781 per 1,000** **(609 to 891)**	**OR 1.19** **(0.52 to 2.73)**	144 (2 studies)	Low	*See below
	23 weeks	749 per 1,000	**737 per 1,000** **(638 to 818)**	**OR 0.94** **(0.59 to 1.51)**	1136 (5 studies)	Low	*See below
	24 weeks	644 per 1,000	**711 per 1,000** **(609 to 797)**	**OR 1.36** **(0.86 to 2.17)**	3369 (5 studies)	Low	*See below
**NEC > Stage II**	22–24 weeks	78 per 1,000	**74 per 1,000** **(60 to 91)**	**OR 0.95** **(0.76 to 1.19)**	5403 (6 studies)	Low	*See below
	22 weeks	66 per 1,000	**40 per 1,000** **(2 to 458)**	**OR 0.59** **(0.03 to 12.03)**	263 (2 studies)	Low	*See below
	23 weeks	89 per 1,000	**83 per 1,000** **(61 to 114)**	**OR 0.93** **(0.66 to 1.32)**	1601 (6 studies)	Low	*See below
	24 weeks	72 per 1,000	**68 per 1,000** **(52 to 90)**	**OR 0.95** **(0.71 to 1.29)**	3539 (4 studies)	Low	*See below

IVH- Intraventricular hemorrhage, PVL- Periventricular leukomalacia, CLD- Chronic lung disease, NEC- Necrotising enterocolitis

OR- Odds ratio, CI: Confidence interval.

*Grading was started as low due to observational nature of all the included studies.

# Evidence upgraded as moderate in view of very large effect size.

## Discussion

Our results indicate that exposure to ANC was associated with significantly reduced mortality and severe IVH/PVL in neonates born <25 weeks of gestation. The effects of ANC were consistent, with no significance difference in neonatal mortality between 22, 23, and 24 weeks. The benefit for severe IVH/PVL was significant only for neonates born at 24 weeks. There was a trend towards benefit for this outcome at 23 weeks. There was no effect of ANC on NEC and CLD. Our findings are in line with the recent systematic review by Park et al, which showed reduced mortality of neonates born at 22 and 23 weeks’ gestation.[12] However, we have also provided data on neonates born at 24 weeks’ gestation which is important to optimise parental counselling and management of such pregnancies given the controversy around ANC at such early gestation.

The uncertainty about ANC before 24 weeks of gestation relates to the developmental immaturity of the lung, and the high risk of mortality and long-term neurodevelopmental impairment. In the context of developmental immaturity, it is important to note that animal and in vitro lung tissue studies have showed that ANC might have beneficial effect on the lungs even before 24 weeks of gestation. [31–37] The mechanisms for such benefits include acceleration of development of type 1 and type 2 pneumocytes, induction of pulmonary beta receptors, and up regulation of gene expression for epithelial sodium channels etc. [31–37]

Physician biases are known to affect the outcomes of neonates born at limits of viability.[38–40] The higher mortality in these neonates may relate to the decision of offering vs. not offering perinatal care including resuscitation at birth and the extent of it if offered. Findings of recent studies indicate that extremely preterm neonates born at border of viability and offered active care can survive without severe complications.[41, 42] A detailed discussion on the influence of physician biases on neonatal survival in such situation is beyond the scope of this review.

We decided to pool the data on severe IVH and PVL as they are associated with adverse neurological outcomes including cerebral palsy.[43, 44] Canterino et al in their retrospective cohort study of 1161 neonates with gestation 24–34 weeks noted that exposure to ANC (Betamethasone) was associated with 56% reduction of risk of PVL with IVH [adjusted odds ratio (aOR)0.44, 95% confidence interval (CI) 0.25, 0.77] and 58% reduction in isolated PVL (aOR 0.42, 95% CI 0.20, 0.88). The data was adjusted for confounding maternal and foetal characteristics.[45] Baud et al in their retrospective cohort study of 883 neonates of gestation 24–31 weeks observed that antenatal exposure to betamethasone but not to dexamethasone was associated with reduced risk of cystic PVL compared with no betamethasone therapy (aOR 0.5; 95% CI, 0.2 to 0.9). The data was adjusted for confounding maternal and foetal characteristics.[46] Kari et al in a RCT of neonates born under 32 weeks’ gestation (N = 79, ANC: 41, Placebo: 38) noted that exposure to ANC (dexamethasone) and postnatal surfactant was associated with significant reduction in IVH and PVL (ANC: 13%; Placebo: 33%; P < 0.01). The incidence of isolated PVL was also reduced but was not statistically significant owing to low power of the study [ANC: 1.3% (1/77) Vs. Placebo: 6.25% (4/64)]. [47] The mechanisms of benefits of ANC in reducing the risk of PVL include enhancement of cytodifferentiation leading to reduced watershed regions in periventricular white matter, accelerated maturation of endothelial cells of the cerebral vasculature and improved auto regulation of cerebral perfusion.[31, 48, 49] ANC is known to reduce the systematic inflammatory response associated with PVL.[50]

The strengths of our review include its robust methodology including the use of appropriate tools for assessing non-RCTs, the large cumulative and gestational age specific sample size, and inclusion of data from a range of nations. We have also summarised the level of evidence as per the GRADE guidelines. The validity and precision of our results is supported by the tight confidence intervals, and the small p values.

Our review provides additional information compared to Park et al.[12] We included neonates born not only at 22 and 23 weeks, but also at 24 weeks’ gestation because the controversy about active intervention including ANC involves this gestational age group. Our review provides additional data from the UK, Brazilian and Swiss neonatal networks. Furthermore, we have pooled the data on severe IVH and PVL to reflect on neurological morbidity.

The limitations include the fact that the data originated from non-RCTs which are prone to biases. We were not able to obtain data from 12 potentially eligible studies despite contacting the authors. The non-availability of individual patient data makes it difficult to adjust for confounding factors such as gender, intrauterine growth restriction, and partial vs. complete course of ANC. We were unable to assess the effects of complete vs. incomplete exposure to ANC using different types of glucocorticoids. Importantly data on long-term neurodevelopment was insufficient. Given the non-RCT design it is difficult to comment on the role of physician biases on the management and outcome of such pregnancies.

### Conclusion

In summary, moderate-low grade evidence suggests that in preterm neonates born <24 weeks, exposure to ANC was associated with significant reduction in mortality and severe IVH/or PVL. Mortality was consistently reduced specifically for gestations 22, 23, and 24 weeks. The benefit for severe IVH/PVL was significant only for neonates born at 24 weeks. Considering the difficult ethical and logistical issues involved in conducting definitive RCTs, data from high quality large non-RCTs and systematic review may be helpful in guiding clinical practice.

## Supporting information

S1 Fig Effects of Antenatal corticosteroids on CLD. (TIF)

S2 Fig Effects Antenatal corticosteroids on NEC. (TIF)

S1 Table Compliance of MOOSE statement. (DOC)

S1 File Supporting information file 1– Search strategy. (DOCX)

S2 File Supporting information file 2- List of excluded studies. (DOCX)
