# Supplementary figures and images for: Antenatal corticosteroids for neonates born before 25 Weeks—A systematic review and meta-analysis
Source: PLoS One. 2017 May 9;12(5):e0176090. doi: 10.1371/journal.pone.0176090 (PMC5423600; doi:10.1371/journal.pone.0176090)

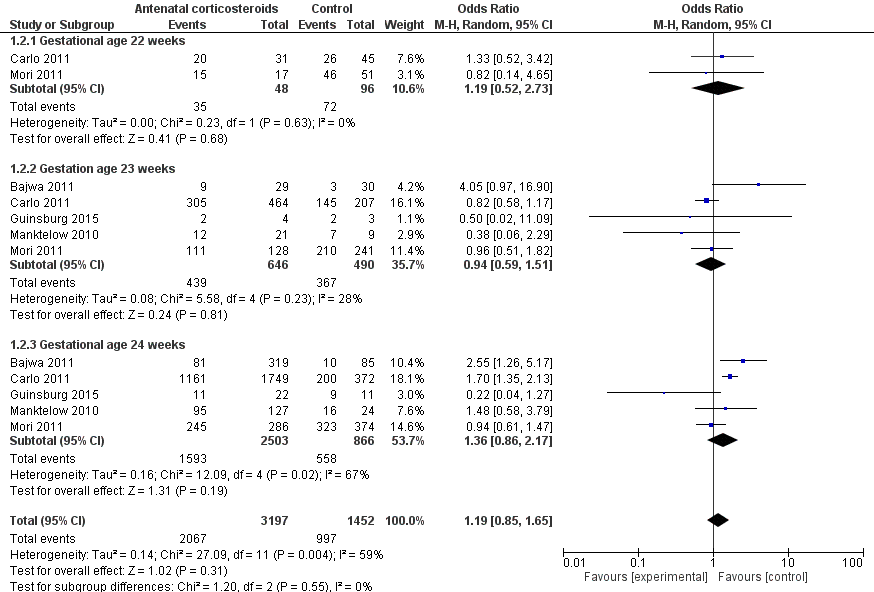

Supplement: S1 Fig — (TIF) [file pone.0176090.s001.tif]

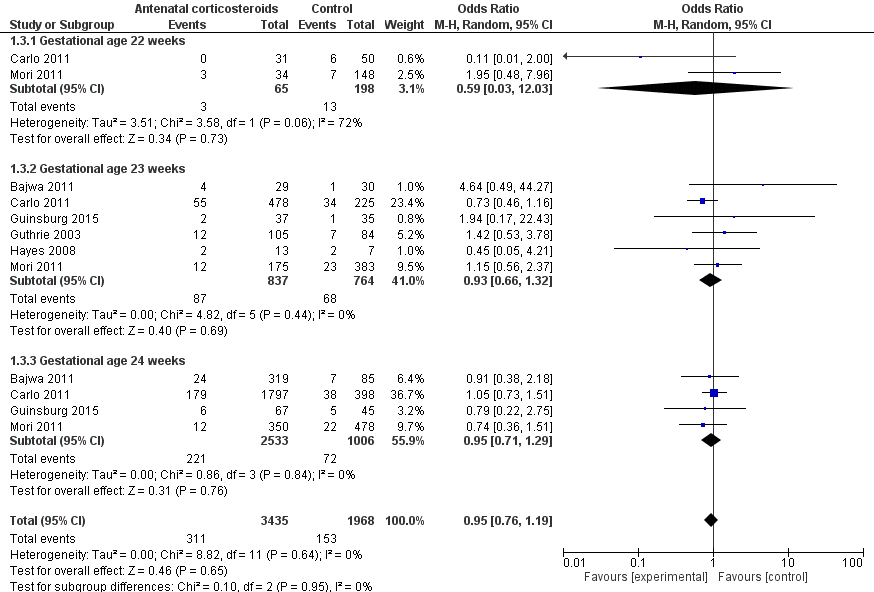

Supplement: S2 Fig — (TIF) [file pone.0176090.s002.tif]
