# Supplementary material for: Antenatal corticosteroids for neonates born before 25 Weeks—A systematic review and meta-analysis
Source: PLoS One. 2017 May 9;12(5):e0176090. doi: 10.1371/journal.pone.0176090 (PMC5423600; doi:10.1371/journal.pone.0176090)
Supplement: S1 File — (DOCX) [file pone.0176090.s004.docx]

**Search Strategies for Antenatal Corticosteroids**

**Search done on 6th February 2017**

Database: **Ovid MEDLINE(R) Epub Ahead of Print, In-Process & Other Non-Indexed Citations, Ovid MEDLINE(R) Daily and Ovid**

**MEDLINE(R) <1946 to Present>**

Search Strategy:

--------------------------------------------------------------------------------

1 antenatal corticosteroid*.mp. (868)

2 adrenal cortex hormones/ (59046)

3 exp glucocorticoids/ (175980)

4 glucocorticoid*.mp. (97352)

5 dexamethasone.mp. (64594)

6 celestone.mp. (70)

7 betamethasone.mp. (7097)

8 antenatal steroid*.mp. (839)

9 adrenal cortex hormone*.mp. (59309)

10 or/1-9 (273505)

11 infant, extremely premature/ (1065)

12 extreme* prematur*.tw. (1470)

13 extreme* preterm.tw. (1579)

14 extreme* low gestation*.tw. (194)

15 (ELGAN or ELGANS).tw. (74)

16 extreme* low birth weight*.tw. (1993)

17 extreme* low birthweight*.tw. (436)

18 exp infant, very low birth weight/ (8544)

19 very premature.tw. (675)

20 very preterm.tw. (2904)

21 very low gestation*.tw. (52)

22 VLBW.tw. (3095)

23 ELBW.tw. (1093)

24 very low birth weight*.tw. (6433)

25 very low birthweight*.tw. (1857)

26 periviable.tw. (80)

27 periviability.tw. (12)

28 or/11-27 (18725)

29 10 and 28 (986)

30 limit 29 to humans (905)

31 exp Cohort Studies/ (1611874)

32 exp case-control studies/ (826854)

33 observational study/ (30132)

34 exp controlled clinical trial/ (533669)

35 controlled clinical trial.pt. (91788)

36 observational study.pt. (30132)

37 randomized controlled trial.pt. (446593)

38 randomi#ed.ab. (464753)

39 placebo.ab. (183731)

40 drug therapy.fs. (1928290)

41 randomly.ab. (270776)

42 (trial or trials).ab. (729950)

43 groups.ab. (1671133)

44 or/31-43 (5323935)

45 30 and 44 (657)

Database: **Embase <1974 to 2017 January 31> Search Strategy:**

--------------------------------------------------------------------------------

1 exp glucocorticoid/ (645467)

2 corticosteroid/ (221802)

3 corticosteroid therapy/ (41255)

4 glucocorticoid*.mp. (117625)

5 dexamethasone.mp. (141482)

6 celestone.mp. (904)

7 betamethasone.mp. (21419)

8 antenatal steroid*.mp. (1313)

9 adrenal cortex hormone*.mp. (1822)

10 or/1-9 (813590)

11 extremely low birth weight/ or very low birth weight/ (13589)

12 extreme*.tw. (288667)

13 Prematurity/ (97200)

14 12 and 13 (6013)

15 extreme* prematur*.tw. (2006)

16 extreme* preterm.tw. (2302)

17 extreme* low gestation*.tw. (244)

18 (ELGAN or ELGANS).tw. (99)

19 extreme* low birth weight*.tw. (2538)

20 extreme* low birthweight*.tw. (516)

21 very premature.tw. (860)

22 very preterm.tw. (3971)

23 very low gestation*.tw. (80)

24 perviable.tw. (0)

25 periviability.tw. (26)

26 VLBW.tw. (4148)

27 ELBW.tw. (1528)

28 or/15-27 (15106)

29 11 or 14 or 28 (22827)

30 10 and 29 (2142)

31 limit 30 to human (2022)

32 cohort analysis/ (313507)

33 exp case control study/ (144662)

34 observational study/ (129428)

35 exp controlled clinical trial/ (647436)

36 randomi#ed.ab. (626016)

37 placebo.ab. (243944)

38 dt.fs. (3367558)

39 ct.fs. (566570)

40 randomly.ab. (345273)

41 (trial or trials).ab. (967562)

42 groups.ab. (2220801)

43 or/32-42 (6523814)

44 31 and 43 (1411)

45 limit 44 to exclude medline journals (93)

46 limit 44 to (conference abstract or conference paper or conference proceeding) (138)

47 or/45-46 (219)

**Cochrane Trials**

#1 MeSH descriptor: [Adrenal Cortex Hormones] this term only 2297

#2 MeSH descriptor: [Dexamethasone] explode all trees 2722

#3 MeSH descriptor: [Glucocorticoids] explode all trees 3973

#4 MeSH descriptor: [Betamethasone] explode all trees 1106

#5 "antenatal corticosteroid*" or glucocorticoid* or dexamethasone* or celeston* or betamethasone or "antenatal steroid*" or "adrenal cortex hormone*" 16100

#6 #1 or #2 or #3 or #4 or #5 16185

#7 MeSH descriptor: [Infant, Extremely Premature] explode all trees 89

#8 "extreme* prematur*" or "extreme* preterm" or "extreme* low birthweight*" or "extreme* low birth weight*" or "very low birthweight*" or "very low birth wieght*" or "very premature" or "very preterm" or "extreme* low gestation*" or "very low gestation*" or VLBW or ELBW or ELGAN or ELGANS or periviable or periviability 2303

#9 #7 or #8 2303

#10 #6 and #9 in Trials 115

**PubMed**

[(antenatal corticosteroid OR antenatal corticosteroids OR antenatal steroid OR antenatal steroids OR adrenal cortex hormone OR adrenal cortex hormones OR glucocorticoid OR glucocorticoids OR dexamethasone OR celestone OR betamethasone) AND (extreme prematurity OR extremely premature OR extreme preterm OR extremely preterm OR extremely low birthweight OR extremely low](https://www.ncbi.nlm.nih.gov/pubmed?term=(antenatal%20corticosteroid%20OR%20antenatal%20corticosteroids%20OR%20antenatal%20steroid%20OR%20antenatal%20steroids%20OR%20adrenal%20cortex%20hormone%20OR%20adrenal%20cortex%20hormones%20OR%20glucocorticoid%20OR%20glucocorticoids%20OR%20dexamethasone%20OR%20celestone%20OR%20betamethasone)%20AND%20(extreme%20prematurity%20OR%20extremely%20premature%20OR%20extreme%20preterm%20OR%20extremely%20preterm%20OR%20extremely%20low%20birthweight%20OR%20extremely%20low%20birthweight%20OR%20extremely%20low%20birth%20weight%20OR%20extremely%20low%20birth%20weights%20OR%20very%20low%20birthweight%20OR%20very%20low%20birthweights%20OR%20very%20low%20birth%20weight%20OR%20very%20low%20birth%20weights%20OR%20very%20premature%20OR%20very%20preterm%20OR%20extremely%20low%20gestational%20OR%20very%20low%20gestational%20OR%20vlbw%20OR%20elbw%20OR%20elgan%20OR%20elegans%20OR%20periviable%20OR%20term%20viability)%20AND%20(pubmednotmedline%5bsb%5d%20OR%20publisher%5bsb%5d%20OR%20inprocess%5bsb%5d)&cmd=correctspelling)**[birthweight](https://www.ncbi.nlm.nih.gov/pubmed?term=(antenatal%20corticosteroid%20OR%20antenatal%20corticosteroids%20OR%20antenatal%20steroid%20OR%20antenatal%20steroids%20OR%20adrenal%20cortex%20hormone%20OR%20adrenal%20cortex%20hormones%20OR%20glucocorticoid%20OR%20glucocorticoids%20OR%20dexamethasone%20OR%20celestone%20OR%20betamethasone)%20AND%20(extreme%20prematurity%20OR%20extremely%20premature%20OR%20extreme%20preterm%20OR%20extremely%20preterm%20OR%20extremely%20low%20birthweight%20OR%20extremely%20low%20birthweight%20OR%20extremely%20low%20birth%20weight%20OR%20extremely%20low%20birth%20weights%20OR%20very%20low%20birthweight%20OR%20very%20low%20birthweights%20OR%20very%20low%20birth%20weight%20OR%20very%20low%20birth%20weights%20OR%20very%20premature%20OR%20very%20preterm%20OR%20extremely%20low%20gestational%20OR%20very%20low%20gestational%20OR%20vlbw%20OR%20elbw%20OR%20elgan%20OR%20elegans%20OR%20periviable%20OR%20term%20viability)%20AND%20(pubmednotmedline%5bsb%5d%20OR%20publisher%5bsb%5d%20OR%20inprocess%5bsb%5d)&cmd=correctspelling)**[OR extremely low birth weight OR extremely low birth weights OR very low birthweight OR very low birthweights OR very low birth weight OR very low birth weights OR very premature OR very preterm OR extremely low gestational OR very low gestational OR vlbw OR elbw OR elgan OR](https://www.ncbi.nlm.nih.gov/pubmed?term=(antenatal%20corticosteroid%20OR%20antenatal%20corticosteroids%20OR%20antenatal%20steroid%20OR%20antenatal%20steroids%20OR%20adrenal%20cortex%20hormone%20OR%20adrenal%20cortex%20hormones%20OR%20glucocorticoid%20OR%20glucocorticoids%20OR%20dexamethasone%20OR%20celestone%20OR%20betamethasone)%20AND%20(extreme%20prematurity%20OR%20extremely%20premature%20OR%20extreme%20preterm%20OR%20extremely%20preterm%20OR%20extremely%20low%20birthweight%20OR%20extremely%20low%20birthweight%20OR%20extremely%20low%20birth%20weight%20OR%20extremely%20low%20birth%20weights%20OR%20very%20low%20birthweight%20OR%20very%20low%20birthweights%20OR%20very%20low%20birth%20weight%20OR%20very%20low%20birth%20weights%20OR%20very%20premature%20OR%20very%20preterm%20OR%20extremely%20low%20gestational%20OR%20very%20low%20gestational%20OR%20vlbw%20OR%20elbw%20OR%20elgan%20OR%20elegans%20OR%20periviable%20OR%20term%20viability)%20AND%20(pubmednotmedline%5bsb%5d%20OR%20publisher%5bsb%5d%20OR%20inprocess%5bsb%5d)&cmd=correctspelling)**[elegans](https://www.ncbi.nlm.nih.gov/pubmed?term=(antenatal%20corticosteroid%20OR%20antenatal%20corticosteroids%20OR%20antenatal%20steroid%20OR%20antenatal%20steroids%20OR%20adrenal%20cortex%20hormone%20OR%20adrenal%20cortex%20hormones%20OR%20glucocorticoid%20OR%20glucocorticoids%20OR%20dexamethasone%20OR%20celestone%20OR%20betamethasone)%20AND%20(extreme%20prematurity%20OR%20extremely%20premature%20OR%20extreme%20preterm%20OR%20extremely%20preterm%20OR%20extremely%20low%20birthweight%20OR%20extremely%20low%20birthweight%20OR%20extremely%20low%20birth%20weight%20OR%20extremely%20low%20birth%20weights%20OR%20very%20low%20birthweight%20OR%20very%20low%20birthweights%20OR%20very%20low%20birth%20weight%20OR%20very%20low%20birth%20weights%20OR%20very%20premature%20OR%20very%20preterm%20OR%20extremely%20low%20gestational%20OR%20very%20low%20gestational%20OR%20vlbw%20OR%20elbw%20OR%20elgan%20OR%20elegans%20OR%20periviable%20OR%20term%20viability)%20AND%20(pubmednotmedline%5bsb%5d%20OR%20publisher%5bsb%5d%20OR%20inprocess%5bsb%5d)&cmd=correctspelling)**[OR periviable OR](https://www.ncbi.nlm.nih.gov/pubmed?term=(antenatal%20corticosteroid%20OR%20antenatal%20corticosteroids%20OR%20antenatal%20steroid%20OR%20antenatal%20steroids%20OR%20adrenal%20cortex%20hormone%20OR%20adrenal%20cortex%20hormones%20OR%20glucocorticoid%20OR%20glucocorticoids%20OR%20dexamethasone%20OR%20celestone%20OR%20betamethasone)%20AND%20(extreme%20prematurity%20OR%20extremely%20premature%20OR%20extreme%20preterm%20OR%20extremely%20preterm%20OR%20extremely%20low%20birthweight%20OR%20extremely%20low%20birthweight%20OR%20extremely%20low%20birth%20weight%20OR%20extremely%20low%20birth%20weights%20OR%20very%20low%20birthweight%20OR%20very%20low%20birthweights%20OR%20very%20low%20birth%20weight%20OR%20very%20low%20birth%20weights%20OR%20very%20premature%20OR%20very%20preterm%20OR%20extremely%20low%20gestational%20OR%20very%20low%20gestational%20OR%20vlbw%20OR%20elbw%20OR%20elgan%20OR%20elegans%20OR%20periviable%20OR%20term%20viability)%20AND%20(pubmednotmedline%5bsb%5d%20OR%20publisher%5bsb%5d%20OR%20inprocess%5bsb%5d)&cmd=correctspelling)**[termviability](https://www.ncbi.nlm.nih.gov/pubmed?term=(antenatal%20corticosteroid%20OR%20antenatal%20corticosteroids%20OR%20antenatal%20steroid%20OR%20antenatal%20steroids%20OR%20adrenal%20cortex%20hormone%20OR%20adrenal%20cortex%20hormones%20OR%20glucocorticoid%20OR%20glucocorticoids%20OR%20dexamethasone%20OR%20celestone%20OR%20betamethasone)%20AND%20(extreme%20prematurity%20OR%20extremely%20premature%20OR%20extreme%20preterm%20OR%20extremely%20preterm%20OR%20extremely%20low%20birthweight%20OR%20extremely%20low%20birthweight%20OR%20extremely%20low%20birth%20weight%20OR%20extremely%20low%20birth%20weights%20OR%20very%20low%20birthweight%20OR%20very%20low%20birthweights%20OR%20very%20low%20birth%20weight%20OR%20very%20low%20birth%20weights%20OR%20very%20premature%20OR%20very%20preterm%20OR%20extremely%20low%20gestational%20OR%20very%20low%20gestational%20OR%20vlbw%20OR%20elbw%20OR%20elgan%20OR%20elegans%20OR%20periviable%20OR%20term%20viability)%20AND%20(pubmednotmedline%5bsb%5d%20OR%20publisher%5bsb%5d%20OR%20inprocess%5bsb%5d)&cmd=correctspelling)**[) AND (pubmednotmedline[sb] OR publisher[sb] OR inprocess[sb])](https://www.ncbi.nlm.nih.gov/pubmed?term=(antenatal%20corticosteroid%20OR%20antenatal%20corticosteroids%20OR%20antenatal%20steroid%20OR%20antenatal%20steroids%20OR%20adrenal%20cortex%20hormone%20OR%20adrenal%20cortex%20hormones%20OR%20glucocorticoid%20OR%20glucocorticoids%20OR%20dexamethasone%20OR%20celestone%20OR%20betamethasone)%20AND%20(extreme%20prematurity%20OR%20extremely%20premature%20OR%20extreme%20preterm%20OR%20extremely%20preterm%20OR%20extremely%20low%20birthweight%20OR%20extremely%20low%20birthweight%20OR%20extremely%20low%20birth%20weight%20OR%20extremely%20low%20birth%20weights%20OR%20very%20low%20birthweight%20OR%20very%20low%20birthweights%20OR%20very%20low%20birth%20weight%20OR%20very%20low%20birth%20weights%20OR%20very%20premature%20OR%20very%20preterm%20OR%20extremely%20low%20gestational%20OR%20very%20low%20gestational%20OR%20vlbw%20OR%20elbw%20OR%20elgan%20OR%20elegans%20OR%20periviable%20OR%20term%20viability)%20AND%20(pubmednotmedline%5bsb%5d%20OR%20publisher%5bsb%5d%20OR%20inprocess%5bsb%5d)&cmd=correctspelling) (337 items)

**Key:**

* = wildcard

# = replaces single character

/ = MeSH term

.ab – words in abstract

exp = explodes the term entered and retrieves records that contain the term and any of its narrower, more specific terms.

.fs = floating subheading

.mp = words in title, abstract, heading word, drug trade name, original title, device manufacturer, drug manufacturer, device trade name, keyword

.tw = words in title or abstract
