# Supplementary material for: Antenatal corticosteroids for neonates born before 25 Weeks—A systematic review and meta-analysis
Source: PLoS One. 2017 May 9;12(5):e0176090. doi: 10.1371/journal.pone.0176090 (PMC5423600; doi:10.1371/journal.pone.0176090)
Supplement: S2 File — (DOCX) [file pone.0176090.s005.docx]

**Total Exclusions: 52**

**Data not suitable for meta- analysis-12**

**Data stratified by gestation and ANC exposure was not available after request. (n=10)**

1.Ancel PY, Goffinet F, Kuhn P, Langer B, Matis J, Hernandorena X, et al. Survival and morbidity of preterm children born at 22 through 34 weeks' gestation in France in 2011: results of the EPIPAGE-2 cohort study. JAMA pediatrics. 2015;169(3):230-8. Epub 2015/01/27. doi: 10.1001/jamapediatrics.2014.3351. PubMed PMID: 25621457.

2.Costeloe K, Hennessy E, Gibson AT, Marlow N, Wilkinson AR. The EPICure study: outcomes to discharge from hospital for infants born at the threshold of viability. Pediatrics. 2000;106(4):659-71. Epub 2000/10/04. PubMed PMID: 11015506.

3.Costeloe KL, Hennessy EM, Haider S, Stacey F, Marlow N, Draper ES. Short term outcomes after extreme preterm birth in England: comparison of two birth cohorts in 1995 and 2006 (the EPICure studies). BMJ (Clinical research ed). 2012;345:e7976. Epub 2012/12/06. doi: 10.1136/bmj.e7976. PubMed PMID: 23212881; PubMed Central PMCID: PMCPMC3514472.

4.Crane JM, Magee LA, Lee T, Synnes A, von Dadelszen P, Dahlgren L, et al. Maternal and perinatal outcomes of pregnancies delivered at 23 weeks' gestation. Journal of obstetrics and gynaecology Canada : JOGC = Journal d'obstetrique et gynecologie du Canada : JOGC. 2015;37(3):214-24. Epub 2015/05/24. PubMed PMID: 26001868.

5.Herber-Jonat S, Schulze A, Kribs A, Roth B, Lindner W, Pohlandt F. Survival and major neonatal complications in infants born between 22 0/7 and 24 6/7 weeks of gestation (1999-2003). American journal of obstetrics and gynecology. 2006;195(1):16-22. Epub 2006/05/09. doi: 10.1016/j.ajog.2006.02.043. PubMed PMID: 16678782.

6.Kollee LA, Cuttini M, Delmas D, Papiernik E, den Ouden AL, Agostino R, et al. Obstetric interventions for babies born before 28 weeks of gestation in Europe: results of the MOSAIC study. BJOG : an international journal of obstetrics and gynaecology. 2009;116(11):1481-91. Epub 2009/07/09. doi: 10.1111/j.1471-0528.2009.02235.x. PubMed PMID: 19583715.

7.Mehler K, Oberthuer A, Keller T, Becker I, Valter M, Roth B, et al. Survival Among Infants Born at 22 or 23 Weeks' Gestation Following Active Prenatal and Postnatal Care. JAMA pediatrics. 2016;170(7):671-7. Epub 2016/05/24. doi: 10.1001/jamapediatrics.2016.0207. PubMed PMID: 27214875.

8.Stoll BJ, Hansen NI, Bell EF, Shankaran S, Laptook AR, Walsh MC, et al. Neonatal Outcomes of Extremely Preterm Infants From the NICHD Neonatal Research Network. Pediatrics. 2010;126(3):443-56. doi: 10.1542/peds.2009-2959.

9.Tyson JE, Parikh NA, Langer J, Green C, Higgins RD. Intensive care for extreme prematurity--moving beyond gestational age. The New England journal of medicine. 2008;358(16):1672-81. Epub 2008/04/19. doi: 10.1056/NEJMoa073059. PubMed PMID: 18420500; PubMed Central PMCID: PMCPMC2597069.

10.El-Metwally D, Vohr B, Tucker R. Survival and neonatal morbidity at the limits of viability in the mid 1990s: 22 to 25 weeks. The Journal of pediatrics. 2000;137(5):616-22. Epub 2000/11/04. doi: 10.1067/mpd.2000.109143. PubMed PMID: 11060525.

**Studies excluded for following reasons related with data:(n=2)**

11.Fernandez R, D'Apremont I, Dominguez A, Tapia JL. Survival and morbidity of very low birth weight infant in a South American neonatal network. Archivos argentinos de pediatria. 2014;112(5):405-12. Epub 2014/09/06. doi: 10.1590/s0325-00752014000500004 10.5546/aap.2014.405. PubMed PMID: 25192520.

The published data about the outcome IVH is available as odds ratio stratified by gestation. Important outcomes such as mortality, CLD, NEC, for neonates born at gestation 22, 23 and 24 weeks based on ANC exposure was not available on request.

12.Wei JC, Catalano R, Profit J, Gould JB, Lee HC. Impact of antenatal steroids on intraventricular hemorrhage in very-low-birth weight infants. Journal of perinatology : official journal of the California Perinatal Association. 2016;36(5):352-6. Epub 2016/03/25. doi: 10.1038/jp.2016.38. PubMed PMID: 27010109; PubMed Central PMCID: PMCPMC4844862.

The article provides data from 24 weeks onwards but was not available in stratified form according to ANC exposure after request.

**Excluded based on selection criteria (n=17)**

13.Abbasi S, Oxford C, Gerdes J, Sehdev H, Ludmir J. Antenatal corticosteroids prior to 24 weeks' gestation and neonatal outcome of extremely low birth weight infants. American journal of perinatology. 2010;27(1):61-6. Epub 2009/06/23. doi: 10.1055/s-0029-1223269. PubMed PMID: 19544249.

14.Althabe F, Belizan JM, Mazzoni A, Berrueta M, Hemingway-Foday J, Koso-Thomas M, et al. Antenatal corticosteroids trial in preterm births to increase neonatal survival in developing countries: study protocol. Reproductive health. 2012;9:22. Epub 2012/09/21. doi: 10.1186/1742-4755-9-22. PubMed PMID: 22992312; PubMed Central PMCID: PMCPMC3477119.

15.Althabe F, Belizan JM, McClure EM, Hemingway-Foday J, Berrueta M, Mazzoni A, et al. A population-based, multifaceted strategy to implement antenatal corticosteroid treatment versus standard care for the reduction of neonatal mortality due to preterm birth in low-income and middle-income countries: the ACT cluster-randomised trial. Lancet (London, England). 2015;385(9968):629-39. Epub 2014/12/03. doi: 10.1016/s0140-6736(14)61651-2. PubMed PMID: 25458726; PubMed Central PMCID: PMCPMC4420619.

16.Amiya RM, Mlunde LB, Ota E, Swa T, Oladapo OT, Mori R. Antenatal Corticosteroids for Reducing Adverse Maternal and Child Outcomes in Special Populations of Women at Risk of Imminent Preterm Birth: A Systematic Review and Meta-Analysis. PloS one. 2016;11(2):e0147604. Epub 2016/02/04. doi: 10.1371/journal.pone.0147604. PubMed PMID: 26841022; PubMed Central PMCID: PMCPMC4740425.

17.Baud O, Foix-L'Helias L, Kaminski M, Audibert F, Jarreau PH, Papiernik E, et al. Antenatal glucocorticoid treatment and cystic periventricular leukomalacia in very premature infants. The New England journal of medicine. 1999;341(16):1190-6. Epub 1999/10/16. doi: 10.1056/nejm199910143411604. PubMed PMID: 10519896.

18.Canterino JC, Verma U, Visintainer PF, Elimian A, Klein SA, Tejani N. Antenatal steroids and neonatal periventricular leukomalacia. Obstetrics and gynecology. 2001;97(1):135-9. Epub 2001/01/12. PubMed PMID: 11152922.

19.Crowther CA, Aghajafari F, Askie LM, Asztalos EV, Brocklehurst P, Bubner TK, et al. Repeat prenatal corticosteroid prior to preterm birth: a systematic review and individual participant data meta-analysis for the PRECISE study group (prenatal repeat corticosteroid international IPD study group: assessing the effects using the best level of evidence) - study protocol. Systematic reviews. 2012;1:12. Epub 2012/05/17. doi: 10.1186/2046-4053-1-12. PubMed PMID: 22588009; PubMed Central PMCID: PMCPMC3351733.

20.Fellman V, Hellstrom-Westas L, Norman M, Westgren M, Kallen K, Lagercrantz H, et al. One-year survival of extremely preterm infants after active perinatal care in Sweden. Jama. 2009;301(21):2225-33. Epub 2009/06/06. doi: 10.1001/jama.2009.771. PubMed PMID: 19491184.

21.Gyamfi-Bannerman C, Thom EA, Blackwell SC, Tita AT, Reddy UM, Saade GR, et al. Antenatal Betamethasone for Women at Risk for Late Preterm Delivery. The New England journal of medicine. 2016;374(14):1311-20. Epub 2016/02/05. doi: 10.1056/NEJMoa1516783. PubMed PMID: 26842679; PubMed Central PMCID: PMCPMC4823164.

22.Kari MA, Hallman M, Eronen M, Teramo K, Virtanen M, Koivisto M, et al. Prenatal dexamethasone treatment in conjunction with rescue therapy of human surfactant: a randomized placebo-controlled multicenter study. Pediatrics. 1994;93(5):730-6. Epub 1994/05/01. PubMed PMID: 8165070.

23.Mwansa-Kambafwile J, Cousens S, Hansen T, Lawn JE. Antenatal steroids in preterm labour for the prevention of neonatal deaths due to complications of preterm birth. International journal of epidemiology. 2010;39 Suppl 1:i122-33. Epub 2010/04/02. doi: 10.1093/ije/dyq029. PubMed PMID: 20348115; PubMed Central PMCID: PMCPMC2845868.

24.Pattanittum P, Ewens MR, Laopaiboon M, Lumbiganon P, McDonald SJ, Crowther CA. Use of antenatal corticosteroids prior to preterm birth in four South East Asian countries within the SEA-ORCHID project. BMC pregnancy and childbirth. 2008;8:47. Epub 2008/10/18. doi: 10.1186/1471-2393-8-47. PubMed PMID: 18925968; PubMed Central PMCID: PMCPMC2596081.

25.Polk DH, Ikegami M, Jobe AH, Sly P, Kohan R, Newnham J. Preterm lung function after retreatment with antenatal betamethasone in preterm lambs. American journal of obstetrics and gynecology. 1997;176(2):308-15. Epub 1997/02/01. PubMed PMID: 9065173.

26.Porto AM, Coutinho IC, Correia JB, Amorim MM. Effectiveness of antenatal corticosteroids in reducing respiratory disorders in late preterm infants: randomised clinical trial. BMJ (Clinical research ed). 2011;342:d1696. Epub 2011/04/14. doi: 10.1136/bmj.d1696. PubMed PMID: 21487057; PubMed Central PMCID: PMCPMC3075234.

27.Romejko-Wolniewicz E, Teliga-Czajkowska J, Czajkowski K. Antenatal steroids: can we optimize the dose? Current opinion in obstetrics & gynecology. 2014;26(2):77-82. Epub 2014/01/28. doi: 10.1097/gco.0000000000000047. PubMed PMID: 24463225; PubMed Central PMCID: PMCPMC3966924.

28.Smith PB, Ambalavanan N, Li L, Cotten CM, Laughon M, Walsh MC, et al. Approach to infants born at 22 to 24 weeks' gestation: relationship to outcomes of more-mature infants. Pediatrics. 2012;129(6):e1508-16. Epub 2012/05/30. doi: 10.1542/peds.2011-2216. PubMed PMID: 22641761; PubMed Central PMCID: PMCPMC3362905.

29.Vogel JP, Souza JP, Gulmezoglu AM, Mori R, Lumbiganon P, Qureshi Z, et al. Use of antenatal corticosteroids and tocolytic drugs in preterm births in 29 countries: an analysis of the WHO Multicountry Survey on Maternal and Newborn Health. Lancet (London, England). 2014;384(9957):1869-77. Epub 2014/08/17. doi: 10.1016/s0140-6736(14)60580-8. PubMed PMID: 25128271.

**Reviews (n=23)**

30.ACOG Obstetric Care Consensus No. 3: Periviable Birth. Obstetrics and gynecology. 2015;126(5):e82-94. Epub 2015/10/22. doi: 10.1097/aog.0000000000001105. PubMed PMID: 26488525.

31.Been JV, Degraeuwe PL, Kramer BW, Zimmermann LJ. Antenatal steroids and neonatal outcome after chorioamnionitis: a meta-analysis. BJOG : an international journal of obstetrics and gynaecology. 2011;118(2):113-22. Epub 2010/11/09. doi: 10.1111/j.1471-0528.2010.02751.x. PubMed PMID: 21054759.

32.Bonanno C, Wapner RJ. Antenatal corticosteroids in the management of preterm birth: are we back where we started? Obstetrics and gynecology clinics of North America. 2012;39(1):47-63. Epub 2012/03/01. doi: 10.1016/j.ogc.2011.12.006. PubMed PMID: 22370107; PubMed Central PMCID: PMCPMC4349395.

33.Campbell DE, Fleischman AR. Limits of viability: dilemmas, decisions, and decision makers. American journal of perinatology. 2001;18(3):117-28. Epub 2001/06/21. doi: 10.1055/s-2001-14530. PubMed PMID: 11414521.

34.Crane J, Armson A, Brunner M, De La Ronde S, Farine D, Keenan-Lindsay L, et al. Antenatal corticosteroid therapy for fetal maturation. Journal of obstetrics and gynaecology Canada : JOGC = Journal d'obstetrique et gynecologie du Canada : JOGC. 2003;25(1):45-52. Epub 2003/01/28. PubMed PMID: 12548324.

35.Crowley P, Chalmers I, Keirse MJ. The effects of corticosteroid administration before preterm delivery: an overview of the evidence from controlled trials. British journal of obstetrics and gynaecology. 1990;97(1):11-25. Epub 1990/01/01. PubMed PMID: 2137711.

36.Gonzales LW, Ballard PL, Ertsey R, Williams MC. Glucocorticoids and thyroid hormones stimulate biochemical and morphological differentiation of human fetal lung in organ culture. The Journal of clinical endocrinology and metabolism. 1986;62(4):678-91. Epub 1986/04/01. doi: 10.1210/jcem-62-4-678. PubMed PMID: 3949950.

37.Hallman M. The Story of Antenatal Steroid Therapy before Preterm Birth. Neonatology. 2015;107(4):352-7. Epub 2015/06/06. doi: 10.1159/000381130. PubMed PMID: 26044103.

38.Haward MF, Kirshenbaum NW, Campbell DE. Care at the edge of viability: medical and ethical issues. Clinics in perinatology. 2011;38(3):471-92. Epub 2011/09/06. doi: 10.1016/j.clp.2011.06.004. PubMed PMID: 21890020.

39.Holsti A, Adamsson M, Serenius F, Hagglof B, Farooqi A. Two-thirds of adolescents who received active perinatal care after extremely preterm birth had mild or no disabilities. Acta paediatrica (Oslo, Norway : 1992). 2016;105(11):1288-97. Epub 2016/06/09. doi: 10.1111/apa.13499. PubMed PMID: 27275954.

40.Lui K, Bajuk B, Foster K, Gaston A, Kent A, Sinn J, et al. Perinatal care at the borderlines of viability: a consensus statement based on a NSW and ACT consensus workshop. The Medical journal of Australia. 2006;185(9):495-500. Epub 2006/12/02. PubMed PMID: 17137454.

41.Onland W, de Laat MW, Mol BW, Offringa M. Effects of antenatal corticosteroids given prior to 26 weeks' gestation: a systematic review of randomized controlled trials. American journal of perinatology. 2011;28(1):33-44. Epub 2010/07/22. doi: 10.1055/s-0030-1262509. PubMed PMID: 20648416.

42.Park CK, Isayama T, McDonald SD. Antenatal Corticosteroid Therapy Before 24 Weeks of Gestation: A Systematic Review and Meta-analysis. Obstetrics and gynecology. 2016;127(4):715-25. Epub 2016/03/10. doi: 10.1097/aog.0000000000001355. PubMed PMID: 26959200.

43.Pignotti MS, Donzelli G. Perinatal care at the threshold of viability: an international comparison of practical guidelines for the treatment of extremely preterm births. Pediatrics. 2008;121(1):e193-8. Epub 2008/01/02. doi: 10.1542/peds.2007-0513. PubMed PMID: 18166538.

44.Rajadurai VS, Tan KH. The use and abuse of steroids in perinatal medicine. Annals of the Academy of Medicine, Singapore. 2003;32(3):324-34. Epub 2003/07/12. PubMed PMID: 12854376.

45.Roberts D, Dalziel S. Antenatal corticosteroids for accelerating fetal lung maturation for women at risk of preterm birth. The Cochrane database of systematic reviews. 2006;(3):Cd004454. Epub 2006/07/21. doi: 10.1002/14651858.CD004454.pub2. PubMed PMID: 16856047.

46.Wapner RJ. Antenatal corticosteroids for periviable birth. Seminars in perinatology. 2013;37(6):410-3. Epub 2013/12/03. doi: 10.1053/j.semperi.2013.06.024. PubMed PMID: 24290397; PubMed Central PMCID: PMCPMC3847668.

47.Zephyrin LC, Hong KN, Wapner RJ, Peaceman AM, Sorokin Y, Dudley DJ, et al. Gestational age-specific risks vs benefits of multicourse antenatal corticosteroids for preterm labor. American journal of obstetrics and gynecology. 2013;209(4):330.e1-7. Epub 2013/06/19. doi: 10.1016/j.ajog.2013.06.009. PubMed PMID: 23770471; PubMed Central PMCID: PMCPMC3967787.

48.Ballard PL, Ballard RA. Scientific basis and therapeutic regimens for use of antenatal glucocorticoids. American journal of obstetrics and gynecology. 1995;173(1):254-62. Epub 1995/07/01. PubMed PMID: 7631700.

49.Obstetric Care Consensus No.4: Periviable Birth.Obstetrics & Gynecology. 2016;127(6):e157-e69.

50. Roberts D. Antenatal corticosteroids to reduce neonatal morbidity and mortality. Green Top guideline. 2010;(7).

51.Antenatal Corticosteroids Clinical Practice Guidelines Panel. Antenatal corticosteroids given to women prior to birth to improve fetal, infant,child and adult health: Clinical Practice Guidelines. Auckland, New Zealand: Liggins Institute, The University of Auckland, 2015.

52. Lee M, Guinn D, Lockwood CJ, Martin R, Brass V. Antenatal corticosteroid therapy for reduction of neonatal morbidity and mortality from preterm delivery; Upto date 2017
